# Digital Detection of Multiple Minority Mutants and Expression Levels of Multiple Colorectal Cancer-Related Genes Using Digital-PCR Coupled with Bead-Array

**DOI:** 10.1371/journal.pone.0123420

**Published:** 2015-04-16

**Authors:** Huan Huang, Shuo Li, Lizhou Sun, Guohua Zhou

**Affiliations:** 1 The First Affiliated Hospital of Nanjing Medical University, Nanjing, China; 2 Nanjing Xiaozhuang University, Nanjing, China; 3 Jinling Hospital, Nanjing University School of Medicine, Nanjing, China; Queen's University Belfast, UNITED KINGDOM

## Abstract

To simultaneously analyze mutations and expression levels of multiple genes on one detection platform, we proposed a method termed “multiplex ligation-dependent probe amplification–digital amplification coupled with hydrogel bead-array” (MLPA–DABA) and applied it to diagnose colorectal cancer (CRC). CRC cells and tissues were sampled to extract nucleic acid, perform MLPA with sequence-tagged probes, perform digital emulsion polymerase chain reaction (PCR), and produce a hydrogel bead-array to immobilize beads and form a single bead layer on the array. After hybridization with fluorescent probes, the number of colored beads, which reflects the abundance of expressed genes and the mutation rate, was counted for diagnosis. Only red or green beads occurred on the chips in the mixed samples, indicating the success of single-molecule PCR. When a one-source sample was analyzed using mixed MLPA probes, beads of only one color occurred, suggesting the high specificity of the method in analyzing CRC mutation and gene expression. In gene expression analysis of a CRC tissue from one CRC patient, the mutant percentage was 3.1%, and the expression levels of CRC-related genes were much higher than those of normal tissue. The highly sensitive MLPA–DABA succeeds in the relative quantification of mutations and gene expressions of exfoliated cells in stool samples of CRC patients on the same chip platform. MLPA–DABA coupled with hydrogel bead-array is a promising method in the non-invasive diagnosis of CRC.

## Introduction

Colorectal cancer (CRC) is the third most common cancer in men and the second most common cancer in women worldwide [1]. In China, CRC is one of the most common malignant cancers and has a high mortality rate. Traditionally, CRC diagnosis uses Dukes classification system, which categorizes the cancer into stages A, B, C, or D. The related data show that 5-year-survival rates of postoperative CRC patients are 81–85% in stage A, 64–78% in stage B, 27–33% in stage C, and 5–14% in stage D; therefore, early diagnosis of CRC and subsequent treatment can effectively increase survival rates. Early screening tests are the key to early diagnosis of CRC and improvement of survival rates [2]. CRC screening tests include procedures such as a colonoscopy, fecal occult blood test, and fibersigmoidoscopy [3]. Although these methods have good clinical results, they also have drawbacks. For example, the invasive techniques might easily cause bleeding and perforation, and the sensitivity and specificity of some methods are insufficient; therefore, the development of a simple, noninvasive approach with high sensitivity and specificity is necessary for the early diagnosis of CRC.

The recent rapid advances in molecular biology make it possible to perform early noninvasive diagnosis of CRC by sensitively analyzing exfoliated cells in the stool samples of CRC patients. Because the oncogenesis of CRC, which involves the interaction of multiple genes, is a multistep process [4], such as activation of proto-oncogenes and inactivation of tumor suppressor genes, the single-gene detection method often misdiagnoses the disease; therefore, the combined detection of multiple genes is helpful in increasing the rate of positive diagnoses of the disease. The occurrence and development of CRC often accompanies gene mutation and changes in gene expression [5]; therefore, CRC diagnosis involves the detection of these activities [6–11]. Although the DNA sequencing technique is a classic method by which gene mutations are detected, it cannot analyze samples in which the number of mutations is <20% [12]. For detecting gene mutations at low levels in the early stages of cancers, an EndoV/ligase-based mutation scanning method was developed with a detection limit of 1.0% [13]; however, the method is incapable of detecting mutants (MUTs) at a much lower level because of its quantitative analysis based on the analog signal. Although the digital analysis method, called BEAMing [14,15], was developed to detect MUTs at an ultra-low level (0.1% of the MUTs), the flow cytometer used in the process is expensive and only one gene is detected in one trial. Because of its high sensitivity and high specificity, quantitative polymerase chain reaction (qPCR) is considered to be the best method by which gene expression can be analyzed; however, because of the limited number of fluorescent markers, qPCR is not suitable for analyzing multiple genes during the same reaction. Although DNA microarray can simultaneously analyze multiple genes, the problem remains of a low detection limit and poor quantitative performance for analyzing cancer-related genes expressed at low levels in the early diagnosis of CRC. Moreover, although the combined analysis of mutations and cancer-related gene expression can increase the accuracy and sensitivity of CRC diagnosis, nearly all the reported techniques apply to only one field of detecting either mutations or gene expression.

Here, we proposed a method termed “multiplex ligation-dependent probe amplification–digital amplification coupled with hydrogel bead-array” (MLPA–DABA) by which mutations and expression levels of multiple genes at low levels can be simultaneously analyzed on one detection platform. Based on previous work [3,16,17], the technical platform of MLPA–DABA was developed by improving the following three aspects: first, MLPA, instead of conventional PCR or target enriched multiplex PCR (Tem-PCR), was used to prepare templates with common ends; second, mutations and expressions of multiple genes, instead of only one or the other, were simultaneously measured with the technical platform by changing the design of specific MLPA probes; third, dye-free and gene-specific MLPA probes, instead of high-cost and gene-specific fluorescent probes, were used to label multiple MUTs and cancer-related genes. The problems of uncertain numbers of dye-labeled deoxynucleotide triphosphates (dNTP) incorporated into a DNA strand and the competition between extension reaction and hybridization reaction referred to in previous works were resolved; therefore, the high specificity, low cost, and high stability of the method were achieved. The method was successfully applied to CRC diagnosis by analyzing the expression of five genes (*β-actin*, *C-myc*, *H-ras*, *CD44v6*, *Cox-2*, and *N-ras*) and three mutation loci on APC genes (codons 1406, 1338, and 1356) in CRC tissues, CRC cells, and stool samples from CRC patients. The results show that the method can be a powerful tool in the early diagnosis of CRC.

## Materials and Methods

### Reagents

N-Hydroxysuccinimide ester (NHS)-activated sepharose HP affinity column and dNTPs were purchased from Amersham Biosciences (Piscataway, NJ). *Taq* DNA polymerase was from Promega (Madison, WI). DC 5225C Formulation Aid and DC 749 Fluid were purchased from Dow Chemical Co. (Midland, MI). Ar20 Silicone Oil was obtained from Sigma (St. Louis, MO). SuperScript III Reverse Transcriptase was purchased from Invitrogen (Carlsbad, CA). Amplicase was purchased from Epicenter (Madison, WI). Other chemicals were of a commercially extra-pure grade. All solutions were prepared with deionized and sterilized water.

### Sequences of primers and probes

The amine-modified primer (5’-NH_2_-TTT TTT TTT TCC ATC TGT TGC GTG CGT GTC-3’) was synthesized by TaKaRa Biotechnology Co., Ltd (Dalian, China). All the other primers were synthesized by Invitrogen Inc. (Shanghai, China). A pair of common primers, common-1 (5’-CCA TCT GTT GCG TGC GTG TC-3’) and common-2 (5’-CCT TGG CAA TCA GGC GAA TC-3’) was used to amplify MLPA products. The gene-specific MLPA probes for detecting mutants were listed in Table 1. The gene-specific MLPA probes for analyzing gene expression were listed in Table 2. The fluorescence probes for bead-decoding were 5’-Cy3-TGC CTT GTC ATT CGG-3’ and 5’-Cy5-GGA AAA GAG CCA A-3’. The reverse-transcription primer was oligo(dT)15.

**Table 1:** Sequences of MLPA probes for mutation detection.

Mutation (codon)	Probe sequences for MLPA
codon 1406	Probe 1: 5’-**CCATCTGTTGCGTGCGTGTC**GTTCGATTGCCAGCTCCGTT-3’
(4216 C > T)	Probe 2: 5’-CAGAGTGAACCATGCAGTGG*TTGGCTCTTTTCC* **GATTCGCCTGATTGC**
	**CAAGG**-3’
	Probe 3: 5’-TAGAGTGAACCATGCAGTGG*CCGAATGACAAGGCA* **GATTCGCCTGATT**
	**GCCAAGG**-3’
codon 1338	Probe 1: 5’-**CCATCTGTTGCGTGCGTGTC**TAGAACCAAATCCAGCAGACTG-3’
(4012 C > T)	Probe 2: 5’-CAGGGTTCTAGTTTATCTTCAGAA*TTGGCTCTTTTCC* **GATTCGCCTGAT**
	**TGCCAAGG**-3’
	Probe 3: 5’-TAGGGTTCTAGTTTATCTTCAGAA*CCGAATGACAAGGCA* **GATTCGCCTG**
	**ATTGCCAAGG**-3’
codon 1356	Probe 1: 5’-**CCATCTGTTGCGTGCGTGTC**GGCACAAAGCTGTTGAATTTTCTT-3’
(4067 C > T)	Probe 2: 5’-CAGGAGCGAAATCTCCCTCC*TTGGCTCTTTTCC* **GATTCGCCTGATTGCC**
	**AAGG**-3’
	Probe 3: 5’-AAGGAGCGAAATCTCCCTCC*CCGAATGACAAGGCA* **GATTCGCCTGATT**
	**GCCAAGG**-3’

Note. The bold bases represent the sequence of common primer. The italic letter bases are source-specific sequence for hybridization with fluorescent probes. The first base at the 5’ end of probe 2 and 3 are a mutation site of interest. The bases with no mark indicate gene-specific sequence.

**Table 2:** Sequences of MLPA probes for gene expression analysis.

Gene symbol	Accession no.	Probe sequences for MLPA
Beta-actin	NM_001101	Probe 1: 5’-**CCATCTGTTGCGTGCGTGTC**CCCAGCACAATGAAGATCAAG-3’
		Probe 2: 5’-ATCATTGCTCCTCCTGAGCG*TTGGCTCTTTTCC* **GATTCGCCTGATT**
		**GCCAAGG**-3’
GAPDH	NM_002046	Probe 1: 5’-**CCATCTGTTGCGTGCGTGTC** CTTTGTCAAGCTCATTTCCTG-3’
		Probe 2: 5’-GTATGACAACGAATTTGGCTAC*CCGAATGACAAGGCA* **GATTCGCC**
		**TGATTGCCAAGG**-3’
C-myc	NM_002467	Probe 1: 5’-**CCATCTGTTGCGTGCGTGTC**GACCACCAGCAGCGACTCTG-3’
		Probe 2: 5’-AGGAGGAACAAGAAGATGAGG*CCGAATGACAAGGCA*G**GATTCGC**
		**CTGATTGCCAAGG**-3’
H-ras	NM_176795	Probe 1: 5’-**CCATCTGTTGCGTGCGTGTC**CGAGGACATCCATCAGTACAG-3’
		Probe 2: 5’-GGAGCAGATCAAGCGGGTC*CCGAATGACAAGGCA*G**GATTCGCCT**
		**GATTGCCAAGG**-3’
CD44v6	BC004372	Probe 1: 5’-**CCATCTGTTGCGTGCGTGTC**GCAACTCCTAGTAGTACAACG-3’
		Probe 2: 5’-GAAGAAACAGCTACCCAGAAG*CCGAATGACAAGGCA*G**GATTCGC**
		**CTGATTGCCAAGG**-3’
Cox-2	NM_000963	Probe 1: 5’-**CCATCTGTTGCGTGCGTGTC**CAGGCAAATTGCTGGCAGG-3’
		Probe 2: 5’-GTTGCTGGTGGTAGGAATGTT*CCGAATGACAAGGCA*G**GATTCGCC**
		**TGATTGCCAAGG**-3’
N-ras	NM_004162	Probe 1: 5’-**CCATCTGTTGCGTGCGTGTC**GTGCGGATATTAACCTCTACAG-3’
		Probe 2: 5’-GGAGCAGATTAAGCGAGTAAA*CCGAATGACAAGGCA*G**GATTCGC**
		**CTGATTGCCAAGG**-3’

Note. The bold bases represent the sequence of common primer. The italic letter bases are source-specific sequence for hybridization with fluorescent probes. The bases with no mark indicate gene-specific sequence.

### Template preparation

Specimens of CRC tissue and CRC cells were obtained from Jiangsu Cancer Hospital (Nanjing, China). The harvesting of tissues, cells and stool from participants was approved by the ethics committee of First Affiliated Hospital of Nanjing Medical University and participants provided written informed consents. Total RNAs were extracted from tissues of CRC patients and CRC cells by using RNeasy Mini Kit (QIAGEN, Germany) according to the manufacturer’s instruction. The extracted RNA was determined by gel electrophoresis performed on a 2% agarose gel and a UV-vis spectrophotometer (Naka Instruments, Japan). The first-strand cDNA was synthesized from oligo(dT)15 primers by using SuperScript III Reverse Transcriptase, according to the manufacturer’s instruction.

Genomic DNA was extracted from tissues and cells by phenol-chloroform extraction. Before extraction, cells were washed twice with normal saline solution and tissues were cut and homogenized in 1 ml of normal saline solution. The extracted DNA was diluted in TE buffer and determined by the UV-vis spectrophotometer.

Stool from a CRC patient in Jiangsu Cancer Hospital (Nanjing, China) was homogenized in ASL buffer and extracted with a QIAamp DNA Stool Mini Kit (Qiagen, Germany).

### Multiplex ligation-dependent probe amplification–digital amplification

After adding the pairs of probes (100 fmol of each) and 1.0 U Ampligase, the reaction mixture containing the DNA or cDNA samples was incubated at 94°C for 1.0 min followed by 60°C for 4.0 min; this cycle was repeated 10 times.

### Digital emulsion PCR (emPCR)

The packed beads (10 μmol NHS sites/ml) from 1 ml of NHS-activated Sepharose HP affinity column were removed from the column and scattered in isopropanol. After washed by 1 mM HCl to thoroughly eliminate isopropanol, the beads were activated by 1 mM ice-cold HCl at 4°C for 1 h. Then, the activated beads and amine-modified primers (10 mM) were incubated in the binding buffer (0.5 M NaCl, 0.2 M NaHCO_3_, pH 7.5) at 20°C for 5 h. The beads were kept away from sedimentation in the incubation. After incubation, the primer-immobilized beads were stored at 4°C for use.

The system of emPCR mixture contains oil phase and aqueous phase. The aqueous phase is divided into two parts, Mock amplification mix and PCR reaction mixture. The ligation products were used as the templates for PCR. First, to make emulsion more stable, 225 μl of mock amplification mix (1×Promega *Taq* Buffer, 2 mM MgCl_2_, 0.1% BSA and 0.01% Tween-80) was homogenized with 375 μl of emulsion oil (40% (w/w) DC 5225C Formulation Aid, 30% (w/w) DC 749 Fluid and 30% (w/w) AR20 Silicone Oil) by a magnetic microstir-bar at 1200 rpm for 5 min in a 5 ml vial. Second, 200 μl of the PCR reaction mix (1×Promega *Taq* Buffer, 2 mM MgCl_2_, 0.5 mM dNTP mixture, 0.125 U/ml *Taq* DNA polymerase, 0.1% BSA, 0.01% Tween-80, 0.06 mM common-2 primer and 0.6 mM each of common-1 primers), primer-coated beads and the templates were added into the vial and then the vial was stirred at 1500 rpm for 3 min to mix the emulsions well. The emulsions were aliquoted with 100 μl each into 8 PCR-tubes. Third, thermocycling was carried out on PTC-225 Peltier Thermal Cycler (MJ RESEARCH, INC.) at an initial denaturation at 95°C for 3 min, then 40 cycles (94°C, 58°C and 68°C for 30 s, 60 s, 90 s each) for amplification and 13 cycles (94°C and 58°C for 30 s, 360 s each) for hybridization and extension.

### Digital bead counting on self-produced hydrogel bead-array

After amplification, the emulsions were broken down by adding isopropanol. The emulsions with isopropanol were filtered through a 25-mm-diameter micropore membrane and the beads were trapped on the membrane. After the beads on the membrane were wished with isopropanol, 80% ethanol in solution (4.0 mM Tris, pH = 7.5), and 0.1% Tween 20, they were recovered in 1.0 mM ethylenediaminetetraacetic acid (EDTA). DNA (dsDNA) immobilized beads were treated with 0.1 mM NaOH for 5.0 min to prepare single-stranded DNA (ssDNA) immobilized beads. After the supernatant was removed, the beads were washed twice with ddH_2_O and stored in the solution.

Acryl-modified slides were prepared according to Xiao et al [18]. The microscope slides (Shanghai Jinglun Industrial Glass Co., Ltd., Shanghai, China) were cleaned by soaking in 10% aqueous nitric acid for 2.0 h.

The hydrogel bead-array was prepared by adding the beads to an acrylamide monomer solution and onto acryl-modified slides and immediately covering with a cover slip (20 × 20 mm) to hold the single-bead layer. After copolymerization, which was carried out at room temperature for 10 min, the cover slip was carefully removed from the hydrogel. The fluorescence probes (1.0 pmol/μL) were hybridized with ssDNA on the beads in the polyacrylamide gel at 40°C for 1.0 h. To remove the free and mismatched probes from the gel chip, the probe-hybridized slides were subjected to electrophoresis at 4.0°C under 50 V for 5.0 min in 1× Tris/borate/EDTA buffer. After washing with water, images of the slides were made using the LuxScanner (CapitalBio Corporation, Beijing, China). Finally, the scanning image of the bead-array was input into Genepix Pro 4.0 to count the color beads on the array.

## Results and Discussion

### Principle

**Fig 1:**
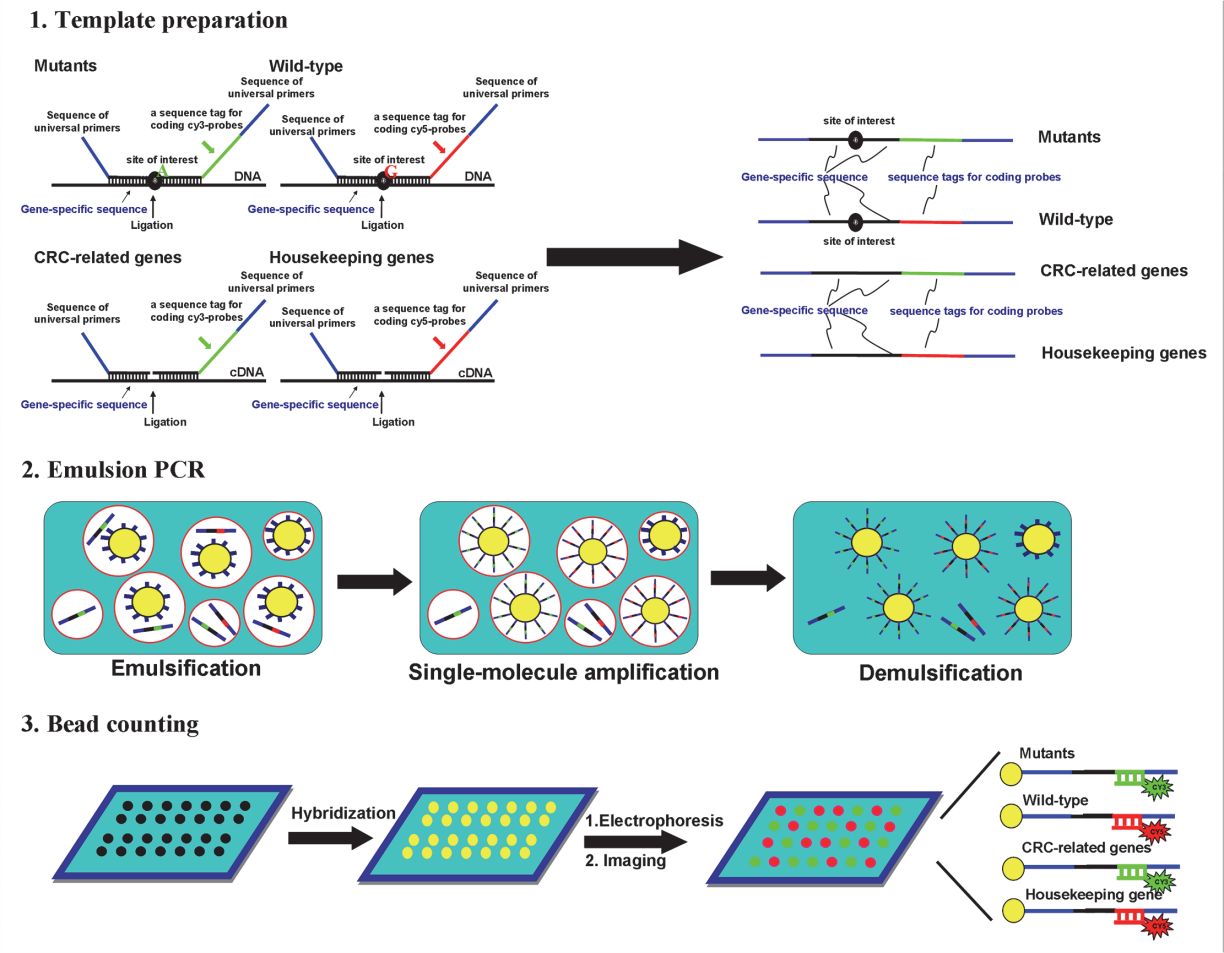
Schematic illustration of multiplex ligation-dependent probe amplification-digital amplification coupled with hydrogel bead-array (MLPA–DABA) for digitally detecting multiple mutants (MUTs) and expressions of multiple colorectal cancer (CRC)-related genes. The assay comprises the following three steps: (1) MLPA for preparing templates tagged with universal sequences; (2) emulsion (em) PCR for preparing beads coated with amplicons generated from a single molecule of MLPA products; and (3) bead decoding by hybridizing universal Cy5-labeled probes and universal Cy3-labeled probes. If a bead displays as green, the amplicons coated on the bead should be amplified from MUT templates or targets of CRC-related genes, while a red bead means wild-type templates or targets of housekeeping genes.

Aimed at simultaneously analyzing multiple mutations and the expression of multiple genes, a target-enriched MLPA was combined with bead-based emPCR for multiplex single-molecule amplification. The protocols of the assay are shown in Fig 1 and comprise the following steps: template preparation, emulsification, single-molecular amplification, demulsification, preparation of hydrogel bead-array, probe hybridization, and bead counting.

First, DNA and cDNA were used as the targets in MLPA to prepare the templates qualified for emPCR, which is an extremely precise digital PCR technique in which there is one reaction for amplifying one molecule to realize single-molecule amplification. In mutation detection, ligation needs three probes for one loci, namely the “left probe”, “right probe 1”, and “right probe 2”. There are two parts in the left probe and three parts in each of the two right probes. The two parts in the left probe are a common tail at the 5ʹ-end for supplying a common priming site needed in the subsequent PCR amplification and a gene-specific sequence at the 3ʹ-end for annealing the target. The right probes contain a gene-specific sequence with a mutation site of interest at the first base of the 5ʹ-end, a source-specific sequence for hybridizing fluorescent probes in the middle, and a common priming site at the 3ʹ-end. Right probes 1 and 2 correspond to the MUT and wild types (WTs), respectively. In gene expression analysis, two probes, left probe and right probe, were used in the ligation reaction. The left probe contains a common priming site and a gene-specific sequence. The right probe contains a gene-specific sequence at the 5ʹ-end, a source-specific sequence in the middle, and a common priming site at the 3ʹ-end. Subsequently, single-molecule amplification was carried out in water-in-oil emulsion. Because both beads and templates from MLPA are diluted so that no more than one target molecule and one bead is present in each compartment, beaded amplicons generated from emPCR originate from exactly one target molecule. After demulsification, beads are collected and fixed as a single-bead layer (40 μm thick) on a hydrogel bead-array. The beads amplified from the housekeeping gene (or WT amplicons) were decoded by hybridizing 3ʹ-Cy5-labeled probes, while the beads amplified from CRC-related genes (or MUTs) were decoded by hybridizing 3ʹ-Cy3-labeled probes; therefore, the number of red (Cy5 dye) beads and the number of green (Cy3 dye) beads reflected the expression levels of the housekeeping gene (or WT amplicons) and CRC-related genes (or MUTs), respectively. The cost of the assay is dramatically decreased by using common fluorescent probes for decoding the beads coated with the amplicons of MUTs, WT amplicons, the housekeeping gene, and CRC-related genes.

### Specificity of bead-array

**Fig 2:**
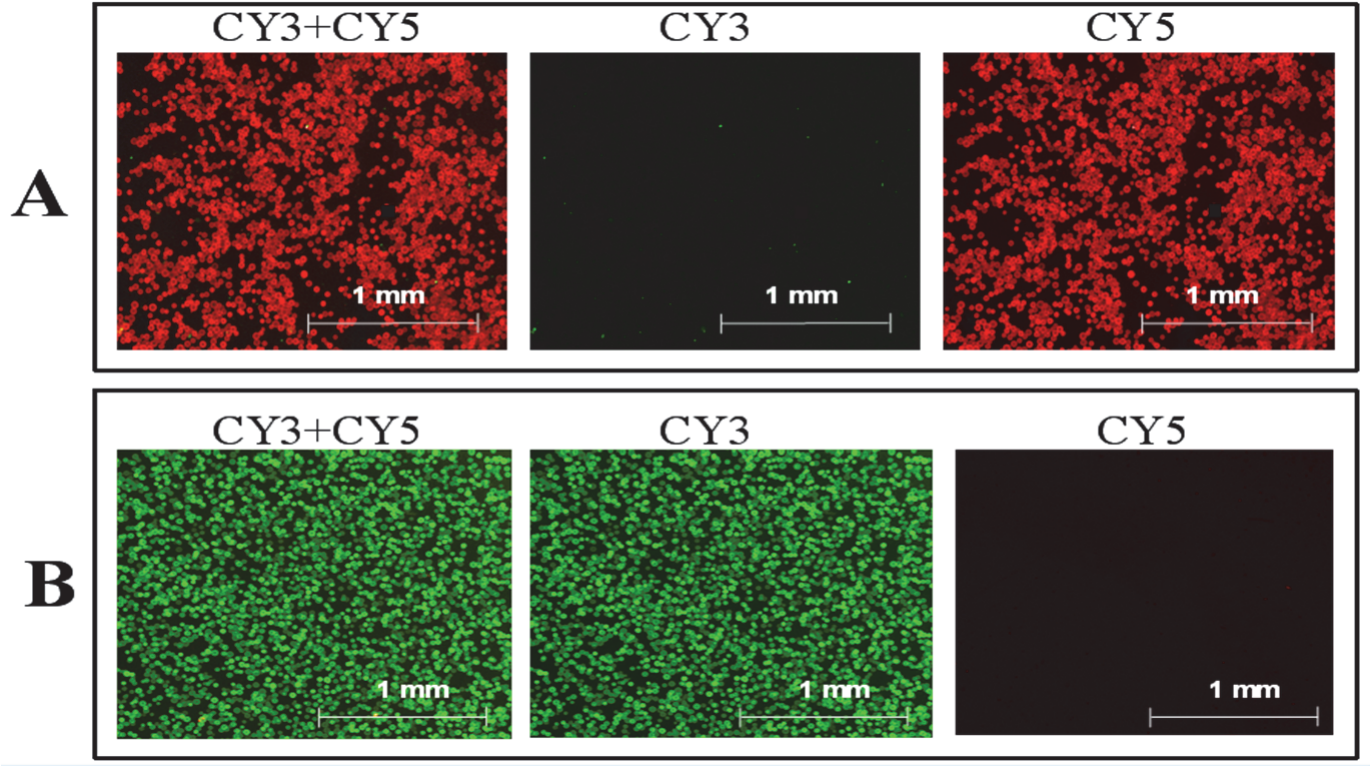
Specificity of hydrogel bead-array for detecting targets, including targets of *β-actin* ligated with multiplex ligation-dependent probe amplification (MLPA) probes containing the sequence tag for coding Cy5-labeled probes (A) and targets of colorectal cancer (CRC)-related genes ligated with MLPA probes containing the sequence tag for coding Cy3-labeled probes (B). Both the Cy5-labeled and Cy3-labeled probes were added to the bead-array for hybridization, and electrophoresis was performed to remove the mismatched and free probes. Finally, the three photographs of panel A or panel B are taken by using three kinds of fluorescence channels, both Cy3 and Cy5 channels, only Cy3 channel, and only Cy5 channel, respectively.

The specificity of the hydrogel chip platform for analyzing the targets from different sources was investigated. The cDNA of *β-actin* was ligated with the gene-specific MLPA probes containing the tag-sequence for coding Cy5-labled probes to produce MLPA products of *β-actin*. The MLPA products were then amplified using two common primers and the 5ʹend of one primer was modified with biotin. After the biotin-modified PCR products were immobilized on streptavidin-modified beads, the bead-array was prepared and covered with Cy5- and Cy3-labled probes for analysis. As shown in Fig 2A, there are only red beads on the images. Similarly, when the cDNA of CRC-related genes was ligated with the gene-specific MLPA probes containing the tag sequence for coding Cy3-labled probes, only green beads can be scanned (Fig 2B). The results indicated that electrophoresis effectively removes the mismatched probes on the beads’ surface or the remaining probes inside the hydrogel. The hybridization between fluorescent probes and targets inside the hydrogel is specific. The platform of the bead-array can be applied to accurately distinguish targets from different sources in MLPA–DABA.

### Specificity of MLPA–DABA for analyzing mutants and gene expressions

**Fig 3:**
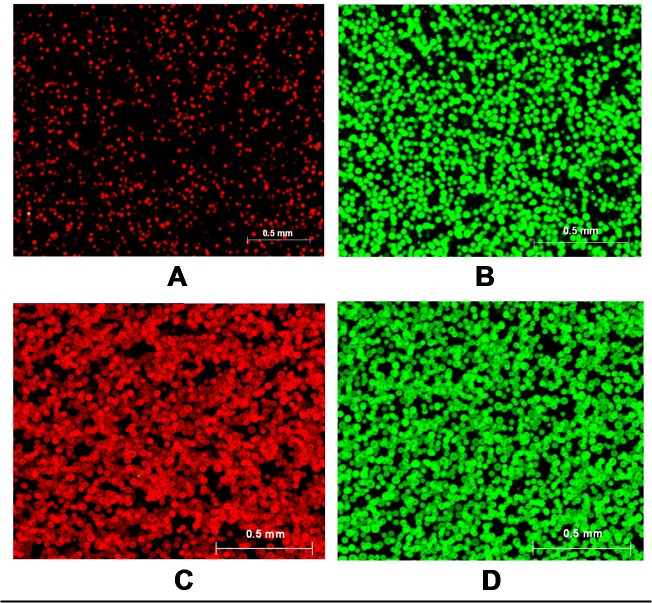
Specificity of multiplex ligation-dependent probe amplification-digital amplification coupled with hydrogel bead-array for detecting gene expressions of colorectal cancer (CRC)-related genes (*cox-2*, *CD44v6*, *C-myc*, *N-ras*, *H-ras*, and *RAB5A*), expression of *β-actin*, mutants (MUTs), and wild-type (WT) targets. The samples were 100% targets of *β-actin* (A); 100% targets of CRC-related genes (B); 100% WT DNA of codon 1338 in normal tissue (C); and 100% MUT DNA of codon 1338 in SW480 cells (D). Seven pairs of probes of CRC-related genes and *β-actin* was added to the MLPA reaction mixture (A and B), and all probes of codons 1406, 1338 and 1356 were used to the MLPA reactions (C and D). After emulsion PCR, both the Cy5-labeled and Cy3-labeled probes were added to the bead-array for hybridization.

To analyze gene expression, the fraction of CRC beads falsely scored in 100% cDNA of *β-actin* and the fraction of housekeeping beads falsely scored in 100% cDNA of CRC genes were determined. After hybridization and electrophoresis, images were taken of the beads coated with 100% fragments from *β-actin* and 100% fragments from CRC-related genes; the results are shown in Fig 3A and 3B. There were nearly no falsely scored beads found in either test; therefore, MLPA–DABA use is highly specific for analyzing expression of CRC-related genes in CRC.

SW480 cells (human CRC cells) with a homozygous mutation at codon 1338 (4012 C > T) were selected as the MUT target. Normal tissue used as the WT target was simultaneously analyzed. The fraction of falsely scored beads in a 100% MUT DNA or in a 100% WT DNA was detected. The results shown in Fig 3C and 3D indicate that the percentage of falsely scored beads (including impurities) in both is nearly 0, suggesting that the method is also specific to mutation detection in CRC.

### Quantification of two genes using MLPA–DABA

**Fig 4:**
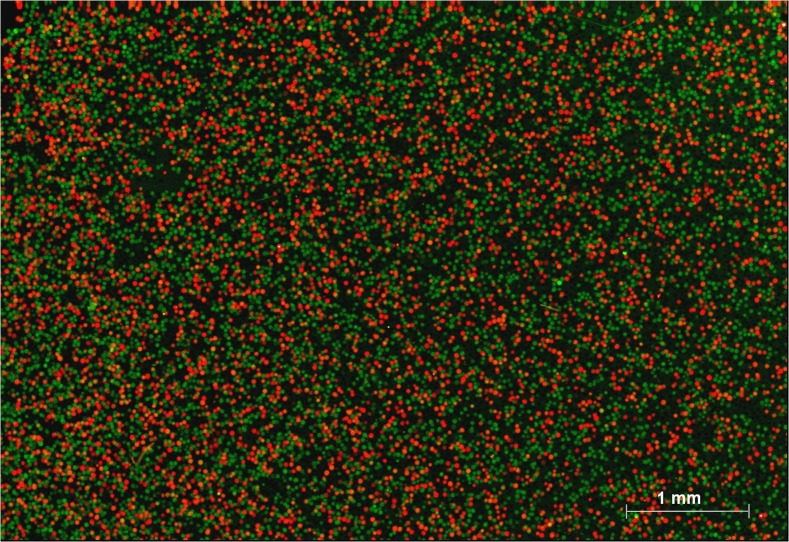
Detection results of a cDNA sample with multiplex ligation-dependent probe amplification-digital amplification coupled with hydrogel bead-array for analyzing expression levels of *β-actin* and *GAPDH*. A red bead originated from one molecule of *β-actin* and a green bead originated from one molecule of *GAPDH*.

To verify the feasibility of using MLPA–DABA to analyze different genes, two housekeeping genes were used as an example. In a tube, the targets of *β-actin* were ligated with the MLPA probes that could hybridize the Cy5-labled probes, while the targets of *GAPDH* were ligated with the MLPA probes that could hybridize the Cy3-labled probes. After digital amplification of ligation products and probe hybridization of the beads, the red and green beads were counted. The results are shown in Fig 4. The number of red beads and green beads reflect the expression levels of *β-actin* and *GAPDH*, respectively. By counting the colored beads on the chip, it was found that the ratio of expression of *β-actin* to that of *GAPDH* was nearly 1:1, suggesting that the two housekeeping genes were successfully detected. There were no yellow beads, indicating that single-molecular amplification was achieved by keeping no more than one target molecule in each compartment, and that it is possible that MLPA–DABA can analyze genes from different sources.

### Application

**Fig 5:**
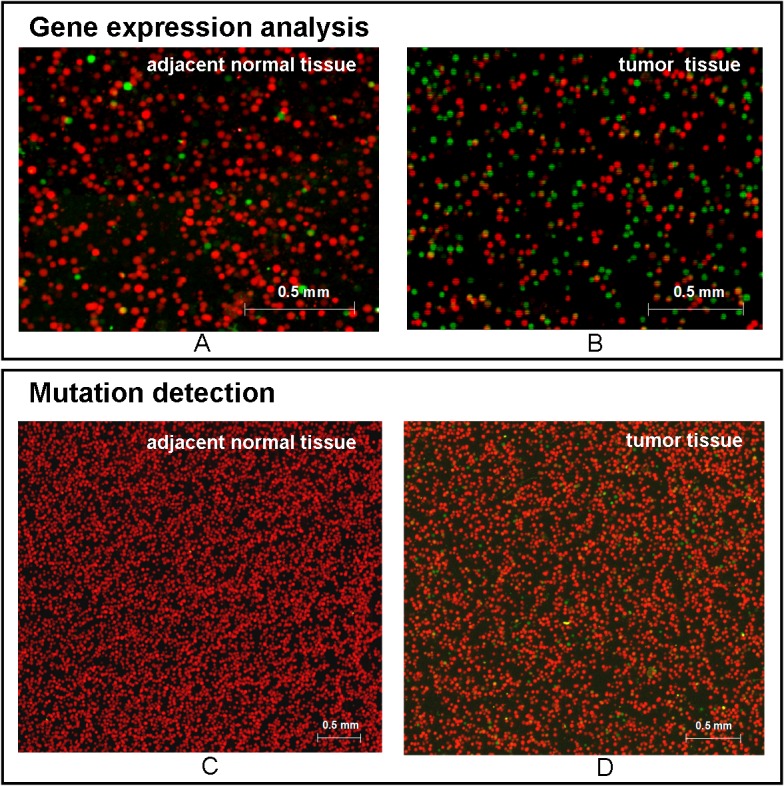
Typical scanned images of bead-array for analyzing adjacent normal tissue and tumor tissue from a colorectal cancer (CRC) patient using multiplex ligation-dependent probe amplification-digital amplification coupled with hydrogel bead-array. (A) Expression analysis of CRC-related genes in adjacent normal tissue; (B) expression analysis of CRC-related genes in tumor tissue; (C) mutation detection of adjacent normal tissue; (D) mutation detection of tumor tissue. Red beads originated from the cDNAs of *β-actin* and wild-type DNA; green beads originated from cDNAs of CRC-related genes (*C-myc*, *H-ras*, *N-ras*, *CD44v6*, and *Cox-2*) and mutant DNA.

To demonstrate the application of MLPA–DABA in the detection of clinical specimens, the relative expression levels of five CRC-related genes and MUTs in both tumor tissues and adjacent normal tissues from CRC patients were detected. The typical scanned images of one patient are shown in Fig 5. The relative expression levels of the total five CRC-related genes to *β-actin* in tumor tissue are much higher than those in the adjacent normal tissue (Fig 5A and 5B). All the beads were red in the DNA analysis of the adjacent normal tissue (Fig 5C), indicating that they were MUT negative, while the green beads (Fig 5D) indicate that they were MUT positive in the tumor tissue. The MUT rate was 3.1%, calculated by the ratio of green beads to red beads. Significant differences in mutation and gene expression between the tumor tissue and the adjacent normal tissue demonstrated that MLPA–DABA can be applied for the early diagnosis of CRC with low-level mutations and a low abundance of gene expression.

**Fig 6:**
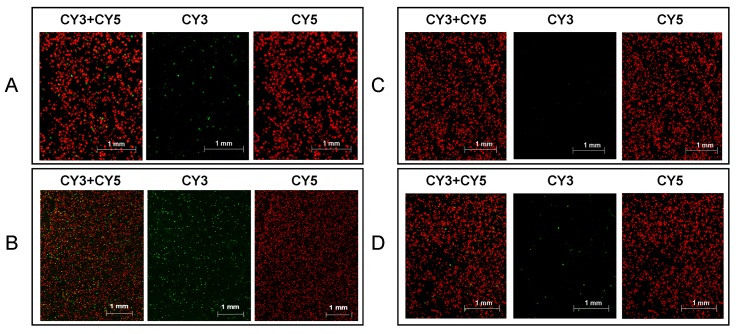
Detection results of stool samples from a colorectal cancer (CRC) patient and a healthy volunteer using multiplex ligation-dependent probe amplification-digital amplification coupled with hydrogel bead-array. (A) Mutation detection of the stool sample from the CRC patient; (B) analysis of CRC-related gene expression of the stool sample from the CRC patient; (C) Mutation detection of the stool sample from the healthy volunteer; (D) analysis of CRC-related gene expression of the stool sample from the healthy volunteer.

To evaluate the feasibility of MLPA–DABA in the noninvasive detection of clinical samples, stools from six CRC patients were used. As shown in S1 Table, only 50% of the six CRC patients (3/6) were MUT positive and 83% of the six CRC patients (5/6) were tested with a high expression of CRC-related genes in noninvasive analyses of stool samples. The total detection rate of MLPA-DABA is 83%. Based on the detection results and the Dukes’ stage of patients, it is concluded that our method is promising in the early diagnosis of colorectal cancer because the detection rate of patients at the early stages (Dukes’ stages A and B) is as high as 75% (3/4). We believe that the detection rate could be further increased by increasing the size of the panel of mutation loci and CRC-related genes. Typical results from MLPA–DABA using one of the stools of the CRC patients and that from a healthy volunteer are shown in Fig 6. The stool from the CRC patient was detected to be MUT positive with a high expression of CRC-related genes, while the stool from the healthy volunteer was detected to be MUT negative with a low expression of CRC-related genes.

## Conclusion

In this study, a sensitive and specific assay for digitally detecting MUTs and expression levels of multiple genes on a hydrogel bead-array was developed. By combining MLPA and bead-based emPCR, single-molecule amplification with common primers used for all targets was achieved. MLPA was applied in the preparation of templates with universal ends from DNA and cDNA samples from different sources, which favors multiplex amplification. Unlike analog amplification in conventional PCR reactions, >10^6^ individual amplification reactions can be carried out simultaneously in one tube with bead-based emPCR.

Beads are immobilized as a single layer on a hydrogel chip to complete high-throughput detection. Compared with the common methods of using acrylamide in electrophoresis to separate proteins and nucleic acids, MLPA–DABA uses polyacrylamide gel to embed the beads. The prepolymer of the acrylamide monomer with beads is dotted on acryl-modified glass and the polymerization is performed to embed the beads on the surface of the glass chip with ammonium persulfate and tetramethylethylenediamine. Because of the high permeability of gels with a 3-dimentional porous structure, the mismatched probes and other impurities embedded in the porous structure can freely pass through the structure; therefore, the probes and impurities can be effectively eliminated by electrophoresis. The high performance of hydrogel overcomes the problems experienced with other methods, such as the high background interference from inadequate washing of the remaining probes. In MLPA–DABA, the background was significantly reduced; subsequently, the sensitivity and specificity of MLPA–DABA were greatly improved. Moreover, because polyacrylamide gel has amphiphilic and uncharged characteristics, the biocompatibility is conducive to hybridization of probes and targets in a solution-like environment.

Because the diagnosis of diseases is made based on the general information of multiple genes, it is not necessary to measure a genetic MUT or the expression of a specific cancer-related gene. A panel of multiple genes as a single diagnostic biomarker demonstrates a more marked difference than individual genes. In MLPA–DABA, one type of fluorescent signal with one color was applied to reflect multiple MUTs and expressions of multiple CRC-related genes. Both general information about the total expression of cancer-related genes and an overall MUT rate are achieved by calculating the ratio of the total number of green beads from various sources to the number of red beads. It is noteworthy that the main steps in the process of MUT detection and the process of gene expression analysis are identical; therefore, MLPA–DABA is capable of analyzing both simultaneously on the same chip platform, which is highly desirable in cancer diagnosis. The use of MLPA–DABA, which has the advantages of being tumor-specific; requiring easy sampling, has great accuracy; is noninvasive to the body; and requires no diet preparation before the test in comparison with other non-invasive methods, such as colonoscopy and fecal occult blood test, is promising for analyzing exfoliated cells in stool samples from CRC patients and will become a powerful tool for non-invasive and early diagnosis of CRC.

## Supporting Information

S1 Table Details and detection results of 6 patients by using stool as starting material. (PDF)
