# Supplementary material for: Digital Detection of Multiple Minority Mutants and Expression Levels of Multiple Colorectal Cancer-Related Genes Using Digital-PCR Coupled with Bead-Array
Source: PLoS One. 2015 Apr 16;10(4):e0123420. doi: 10.1371/journal.pone.0123420 (PMC4399940; doi:10.1371/journal.pone.0123420)
Supplement: S1 Table — (PDF) [file pone.0123420.s001.pdf]

Table S1. Details and detection results of 6 patients by using stool as starting material.

Patient No.	Age	Dukes' stage	Mutation detection	CRC-related gene expression analysis
1	48	D	positive	↑
2	60	B	negative	↑
3	54	B	positive	↑
4	45	B	negative	↓
5	68	C	positive	↑
6	58	A	negative	↑

" ↑ " represents "the patient with a high expression of CRC-related genes".

" ↓ " represents "the patient with a low expression of CRC-related genes".
